# The effect of ultrasound-guided erector spinae plane block on postsurgical pain: a meta-analysis of randomized controlled trials

**DOI:** 10.1186/s12871-020-01016-8

**Published:** 2020-05-01

**Authors:** Mark C. Kendall, Lucas Alves, Lauren L. Traill, Gildasio S. De Oliveira

**Affiliations:** Department of Anesthesiology, The Warren Alpert Medical School of Brown University, Providence, Rhode Island USA

**Keywords:** Erector spinae plane block, Postoperative pain, GRADE criteria, Meta-analysis

## Abstract

**Background:**

The effect of erector spinae plane block has been evaluated by clinical trials leading to a diversity of results. The main objective of the current investigation is to compare the analgesic efficacy of erector spinae plane block to no block intervention in patients undergoing surgical procedures.

**Methods:**

We performed a quantitative systematic review of randomized controlled trials in PubMed, Embase, Cochrane Library, and Google Scholar electronic databases from their inception through July 2019. Included trials reported either on opioid consumption or pain scores as postoperative pain outcomes. Methodological quality of included studies was evaluated using Cochrane Collaboration’s tool.

**Results:**

Thirteen randomized controlled trials evaluating 679 patients across different surgical procedures were included. The aggregated effect of erector spinae plane block on postoperative opioid consumption revealed a significant effect, weighted mean difference of − 8.84 (95% CI: − 12.54 to − 5.14), (*P* < 0.001) IV mg morphine equivalents. The effect of erector spinae plane block on post surgical pain at 6 h compared to control revealed a significant effect weighted mean difference of − 1.31 (95% CI: − 2.40 to − 0.23), *P* < 0.02. At 12 h, the weighted mean difference was of − 0.46 (95% CI: − 1.01 to 0.09), *P* = 0.10. No block related complications were reported.

**Conclusions:**

Our results provide moderate quality evidence that erector spinae plane block is an effective strategy to improve postsurgical analgesia.

## Background

The misuse of prescribed opioids leading to the current opioid epidemic crisis has put greater emphasis on the development of non-opioid analgesic techniques to manage postoperative pain [1–3]. A large variety of regional anesthesia techniques have been commonly used to minimize postoperative pain [4–6]. In addition, several techniques (e.g., transverse abdominis plane blocks, pectoral nerve blocks, brachial plexus blocks) have been evaluated in quantitative systematic reviews [7–9]. These techniques have emerged as effective non-opioid strategies to reduce post-surgical pain.

The erector spinae plane block has been used clinically by anesthesiologists as a potential non-opioid analgesic strategy across multiple surgical procedures [10–14]. The block is considered easy to perform and can be easily implemented in the perioperative period [15, 16]. Recent clinical trials have assessed the efficacy of the erector spinae plane block on postoperative analgesia with inconsistent results. Nonetheless, to the best of our knowledge, no quantitative systematic review has evaluated the effectiveness of the erector spinae plane block to improve postoperative analgesia.

The objective of our study was to examine the analgesic efficacy of erector spinae plane block for postoperative analgesia outcomes in patients undergoing surgical procedures. In addition, we also investigated the potential side effects related to the use of the erector spinae plane block.

## Methods

We performed a quantitative systematic review according to the PRISMA guidelines [17]. The study was registered with the PROSPERO international database (CRD42020148072; registered August 2019). We followed similar methods as previously published by our group [18, 19].

### Systematic search and inclusion criteria

A comprehensive search of randomized trials investigating the effects of erector spinae plane block to control (i.e. no block or sham block) on postoperative surgical analgesia was performed using web-based electronic databases PubMed, Google Scholar, the Cochrane Database of Systematic Reviews, and Embase from inception up to July 2019. The search words ‘erector spine block’, ‘erector spinae plane block’, or “ESPB” were used in various combinations using Boolean operators. Search strategy is shown in Additional file 1. The search was limited to adults older than 18 years of age and there were no language restrictions. The bibliographies of the identified studies were evaluated and reviewed for additional studies. There was no search performed for unpublished or non-peer reviewed studies. Included trials reported either opioid consumption or pain scores as postoperative pain outcomes. No minimum sample size was required for inclusion in the quantitative analysis.

### Exclusion criteria

Studies were excluded if a direct comparison of erector spinae plane block and no block could not be determined. Non-randomized controlled trials, anatomical or cadaver studies, case reports, or editorials were not considered for inclusion.

### Selection of included studies and data extraction

Two investigators (MCK and LA) independently reviewed the abstracts obtained from the initial search using the predetermined inclusion and exclusion criteria. The trials that were not relevant based on the inclusion criteria were omitted. Any discrepancies encountered during the selection process were resolved by discussion among the evaluators (MCK and LA). If there was a disagreement then the final decision was determined by the senior investigator (GDO). Data extraction was carried out by using a pre-designed data collection form. The primary source of data extraction was from either the text or tables. If the data could not be found in either location, we extracted the data manually from available figures or plots. The extracted data obtained from studies included the sample size, number of study participants in treatment/control groups, surgery description, type of local anesthetic dose, single-shot or bilateral block placement, use of ultrasonography for block placement, postoperative opioid consumption, postoperative pain scores, postoperative nausea and vomiting, and adverse events. Postoperative opioid consumption was converted to intravenous morphine milligram equivalents assuming no cross-tolerance (morEq) [20]. Continuous data was recorded using mean and standard deviation. Data variables presented as median, interquartile range, or mean ± 95% confidence interval (CI) were transformed to mean and standard deviation [21, 22]. For studies that did not provide standard deviation, the standard deviation was estimated using the most extreme values. If the same outcome variable was reported more than once then the most conservative value was used.

### Risk of bias assessment

The validity of the included studies was evaluated in accordance with Cochrane risk-of-bias tool (RoB-2) [23]. This recently new assessment tool consists of five domains focusing on where bias might be introduced into a trial. The domains consist of: bias arising from the randomization process, bias due to deviations from the intended interventions, bias due to missing outcome data, bias in measurement of the outcome, and bias in the selection of the reported result. Each category was recorded as “low risk of bias”, “some concerns”, or “high risk of bias.” Two investigators (MCK and LA) independently assessed the risk of bias of included studies and any inconsistencies were resolved by discussion with senior author (GDO).

### Primary outcome

Postoperative opioid consumption (IV morEq) reported at 24 h following surgery.

### Secondary outcomes

Postoperative pain scores (numeric pain rating score, 0 = no pain, 10 = extreme pain) at rest and with activity at 6 h, at 12 h, and at 24 h after surgery, block complications, and postoperative nausea and vomiting displayed as (n).

### Meta-analysis

The weighted mean differences (WMD) with 95% confidence interval (CI) were calculated and reported for continuous data for total opioid consumption up to 24 h and pain scores (NRS) at 6 h, 12 h and at 24 h. Statistical significance required that the 95% CI for continuous data did not include zero and for dichotomous data, the 95% confidence interval did not include 1.0. Due to the variety of surgical procedures, we chose to use the random-effects model in an attempt to generalize our findings to studies not included in our meta-analysis [24]. Asymmetric funnel plots were analyzed for publication bias using Egger’s regression test [25]. A one sided *P* < 0.05 was considered as an indication of an asymmetric funnel plot. In the presence of an asymmetric funnel plot, a file drawer analysis was performed, which estimates the lowest number of additional studies that if they would become available, it would reduce the combined effect to non-significance assuming the average z-value of the combined *P* values of these missing studies would be 0. Heterogeneity was considered to be high if the I^2^ statistic was greater than 50%. If heterogeneity was high, we performed a sensitivity analysis by removing individual studies and investigated surgical procedures by examining its effect on the overall heterogeneity. A *P* value < 0.05 was required to reject the null hypothesis. Analyses was performed using Stata version 15 (College Station, Texas) and Comprehensive Meta-analysis software version 3 (Biostat, Englewood, NJ).

## Results

**Fig. 1:**
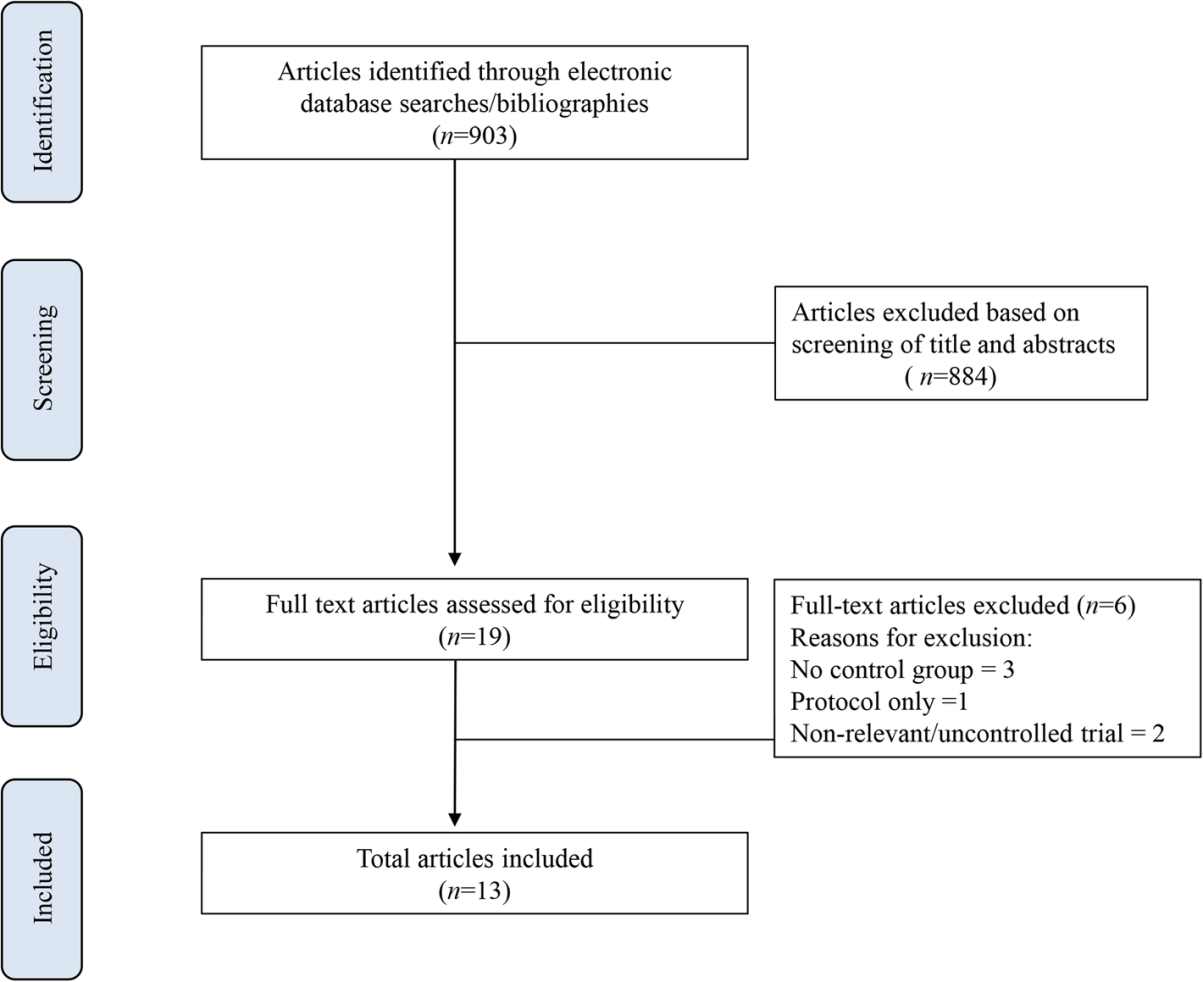
Flow chart of the selection of studies

A flow diagram of the literature search and reasons for exclusion are shown in Fig. 1.

The initial query identified 903 articles and 884 articles were excluded after review of the study abstracts. A total of 19 articles were evaluated and after full-text review 6 articles were omitted. Thirteen studies involving 679 subjects fulfilled the inclusion criteria and were included in the final analysis [26–38]. The median number of patients was 50 (IQR, 40 to 60). All included studies reported on opioid consumption and/or pain scores at rest or during activity. Table 1 provides details of the study characteristics of the included trials.

**Table 1:** Cochrane risk-of-bias assessment for included studies (RoB 2)

Authors/Year	Bias arising from the randomization process	Effect of assignment to intervention	Effect of adhering to intervention	Bias due to missing outcome data	Bias in measurement of outcomes	Bias in the selection of the reported result	Overall risk of bias
Abu Elyazed et al. [26] 2019	Low	Low	Low	Low	Low	Low	Low
Aksu et al. [27] 2019	Low	Some concerns	Low	Low	Low	Low	Some concerns
Ciftci et al. [28] 2018	Low	Some concerns	Low	Low	Some concerns	Low	High
Gurkan et al. [29] 2018	Low	Some concerns	Low	Low	Low	Low	Some concerns
Hamed et al. [30] 2019	Low	Low	Low	Low	Low	Low	Low
Krishna et al. [31] 2019	Low	Low	Low	Low	Low	Low	Low
Oksuz et al. [32] 2019	Low	Some concerns	Low	Low	Low	Low	Some concerns
Singh et al. [33] 2019	Low	Some concerns	Low	Low	Some concerns	Low	High
Singh et al. [34] 2019	Low	Some concerns	Low	Low	Low	Low	Some concerns
Tulgar et al. [35] 2018	Low	Low	Low	Low	Low	Low	Low
Tulgar et al. [36] 2018	Low	Low	Low	Low	Low	Low	Low
Tulgar et al. [37] 2019	Low	Low	Low	Low	Low	Low	Low
Yayik et al. [38] 2019	Some concerns	Some concerns	Low	Low	Low	Low	High

### Quality assessment

All included trials reported inclusion and exclusion criteria and described baseline characteristics. The risk of bias assessment according to the Cochrane Handbook using the Cochrane risk-of-bias assessment tool (RoB-2) is presented in Table 2. The quality of evidence of the included studies was summarized using the Grading of Recommendations, Assessment, Development, and Evaluation (GRADE) criteria and is presented in Table 3.

**Table 2:** Summary of study characteristics included in analysis

Authors	Year of Publication	Procedures	Treatment/Control	UG	Treatment	Anesthesia	Method of extraction
Abu Elyazed et al. [26]	2019	Open epigastic hernia repair	30/30	Y	Bilateral 20 ml 0.25% bupivacaine Sham block (1 ml NS)	General	Text Table
Aksu et al. [27]	2019	Breast surgery	25/25	Y	Single-shot 20 ml 0.25% bupivacaine No block	General	Text Table
Ciftci et al. [28]	2019	Video assisted thoracic surgery	30/30	Y	Single-shot 20 ml 0.25% bupivacaine No block	General	Text Table
Gurkan et al. [29]	2018	Breast cancer surgery	25/25	Y	Single-shot 20 ml 0.25% bupivacaine Sham block (NS)	General	Text Table
Hamed et al. [30]	2019	Abdominal hysterectomy	30/30	Y	Bilateral 20 ml 0.5% bupivacaine Sham block (NS)	General	Text Table
Krishna et al. [31]	2018	Cardiac surgery	53/53	Y	Bilateral 3 mg/kg 0.375% Ropivacaine No block	General	Text Table
Oksuz et al. [32]	2019	Reduction mammoplasty	21/22	Y	Bilateral 20 ml 0.25% bupivacaine No block	General	Text
Singh et al. [33]	2019	Radical mastectomy	20/20	Y	Single-shot 20 ml 0.5% bupivacaine No block	General	Text
Singh et al. [34]	2019	Lumbar spine surgery	20/20	Y	Bilateral 20 ml 0.5% bupivacaine No block	General	Text Table
Tulgar et al. [35]	2019	Laparoscopic Cholecystectomy	20/20	Y	Bilateral 20 ml 0.5% bupivacaine No block	General	Text Table
Tulgar et al. [36]	2018	Orthopedic surgery	20/20	Y	Single-shot 20 ml 0.5% bupivacine No block	General	Text Table
Tulgar et al. [37]	2018	Laparoscopic Cholecystectomy	15/15	Y	Bilateral 20 ml 0.375% bupivacaine No block	General	Text Table
Yayik et al. [38]	2019	Lumbar decompression surgery	30/30	Y	Bilateral 20 ml 0.25% bupivacaine No block	General	Text Table

*UG* ultrasound guided, *NS* normal saline

**Table 3:** Summary of the quality of evidence (GRADE) for comparing erector spinae plane block to a control group for the primary and secondary outcomes of the included studies

# studies in design (n)	Risk of bias	Inconsistency	Indirectness	Imprecision	Publication bias	Overall quality of evidence^e^	Importance
**Postoperative opioid consumption at 24 h**							
12 (573)	None serious^a^	Serious^b^	None serious	None serious	Undetected	⨁⨁⨁◯ Moderate	Important
**Postoperative pain at rest at 6 h**							
9 (486)	None serious^a^	Serious^b^	None serious	None serious	Detected^c^	⨁⨁⨁◯ Moderate	Important
**Postoperative pain at rest at 12 h**							
10 (546)	None serious^a^	Serious^b^	None serious	None serious	Undetected	⨁⨁⨁◯ Moderate	Important
**Postoperative pain at rest at 24 h**							
10 (500)	None serious^a^	Serious^d^	None serious	None serious	Undetected	⨁⨁⨁◯ Moderate	Important
**Postoperative nausea and vomiting**							
11 (596)	None serious^a^	None serious	None serious	None serious	Undetected	⨁⨁⨁⨁ High	Important

^a^Majority of studies had allocation concealment and used blinded outcome assessments; lost to follow up was very low; the overall risk of bias was felt to be none serious

^b^There is high heterogeneity among the included studies; sensitivity analysis did not significantly reduce heterogeneity

^c^Funnel plot did reveal asymmetry; Egger’s test, *P* = < 0.05

^d^There is high heterogeneity among the included studies; subgroup analysis of type of block placement did significantly reduce heterogeneity

^e^Grade Workshop Group grades of evidence: high quality: further research very unlikely to change confidence in estimate of effect; moderate quality; further research likely to have important impact on confidence in estimate of effect and may change estimate; low quality; further research very likely to have important impact on confidence in estimate of effect and likely to change estimate; very low quality: very uncertain about estimate

### Postoperative opioid consumption reported up to 24 h following surgery

**Fig. 2:**
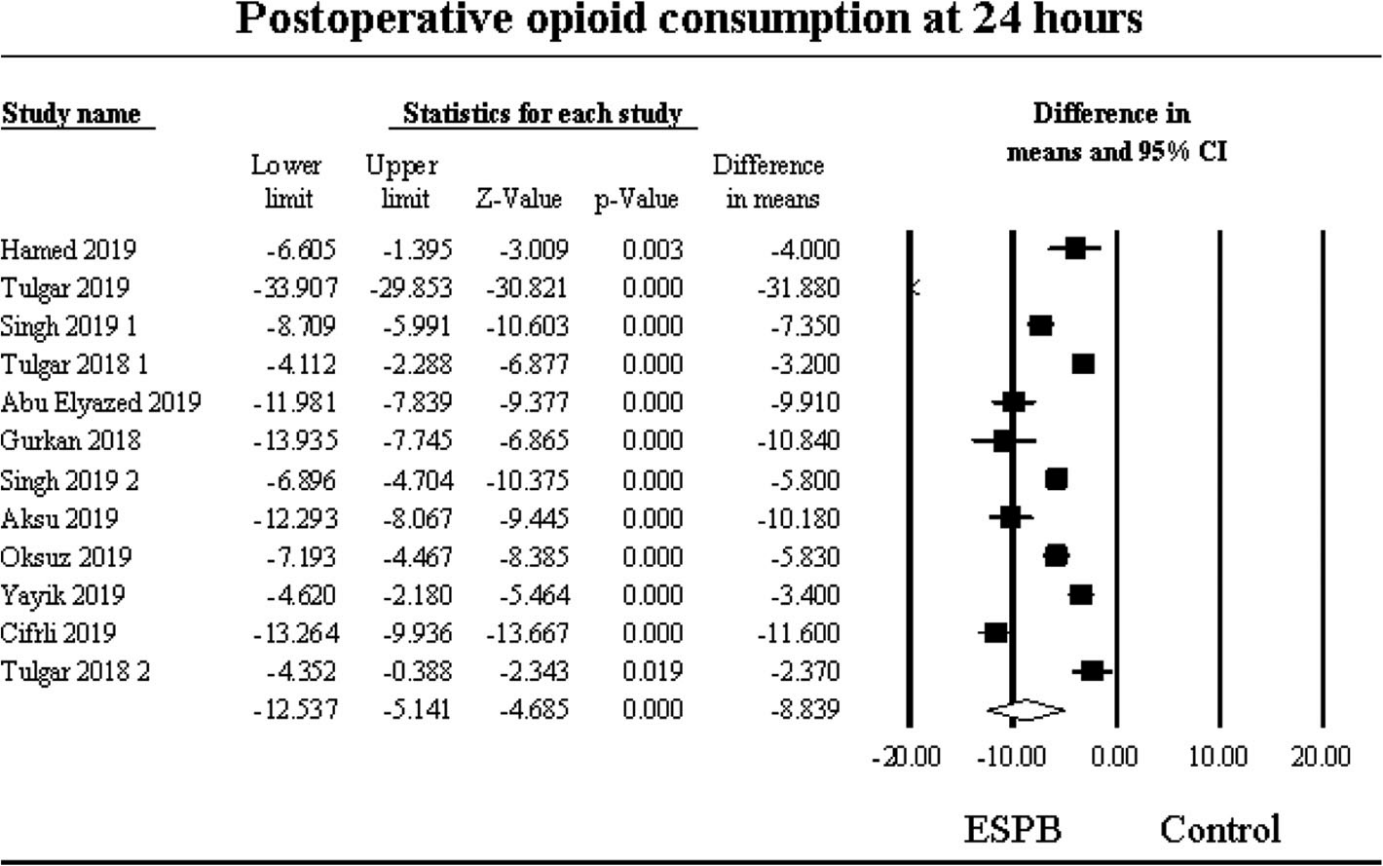
Postoperative opioid consumption at 24 h. Meta-analysis evaluating the effect of erector spinae plane block on opioid consumption compared to control at 24 h following surgery. The overall effect of the erector spinae plane block versus control was estimated as a random effect. The point estimate for the overall effect was − 8.84 (95%CI: − 12.54 to − 5.14), (*P* < 0.001) mg IV morphine equivalents. The weighted mean difference for individual studies is represented by the square symbol on Forrest plot, with 95% CI of the difference shown as a solid line

The pooled effect of twelve studies [26–30, 32–38] examining the effect of erector spinae plane block on postoperative opioid consumption compared to control at 24 h after surgery revealed a significant effect, weighted mean difference (WMD) of − 8.84 (95% CI: − 12.54 to − 5.14), (*P* < 0.001) mg IV morEq (Fig. 2). The heterogeneity was high (I^2^ = 98%) and could be partially explained by whether the block was placed bilaterally or as a single-shot procedure (I^2^ = 91%). The type of surgery did not substantially reduce the heterogeneity any further (I^2^ = 86%). Potential sources of heterogeneity were further tested by a sensitivity analysis by removing individual studies which did not significantly reduce the heterogeneity among the studies.

A subgroup analysis of surgery type revealed the reduction of opioid consumption compared to control was statistically significant in patients who underwent chest surgical procedures WMD of − 9.04 (95% CI: − 11.37 to − 6.70), *P* < 0.001 and in patients who underwent spine or orthopedic procedures WMD of − 4.13 (95% CI: − 5.78 to − 2.48), *P* < 0.001. Patients who had abdominal surgery did not experience statistical significance, WMD of − 12.05 (95% CI: − 25.88 to 1.79), *P* = 0.09. Visual examination of the funnel plot and Egger’s regression test (*P* = 0.06) revealed no apparent publication bias.

### Postoperative pain at rest 6 h after surgery

**Fig. 3:**
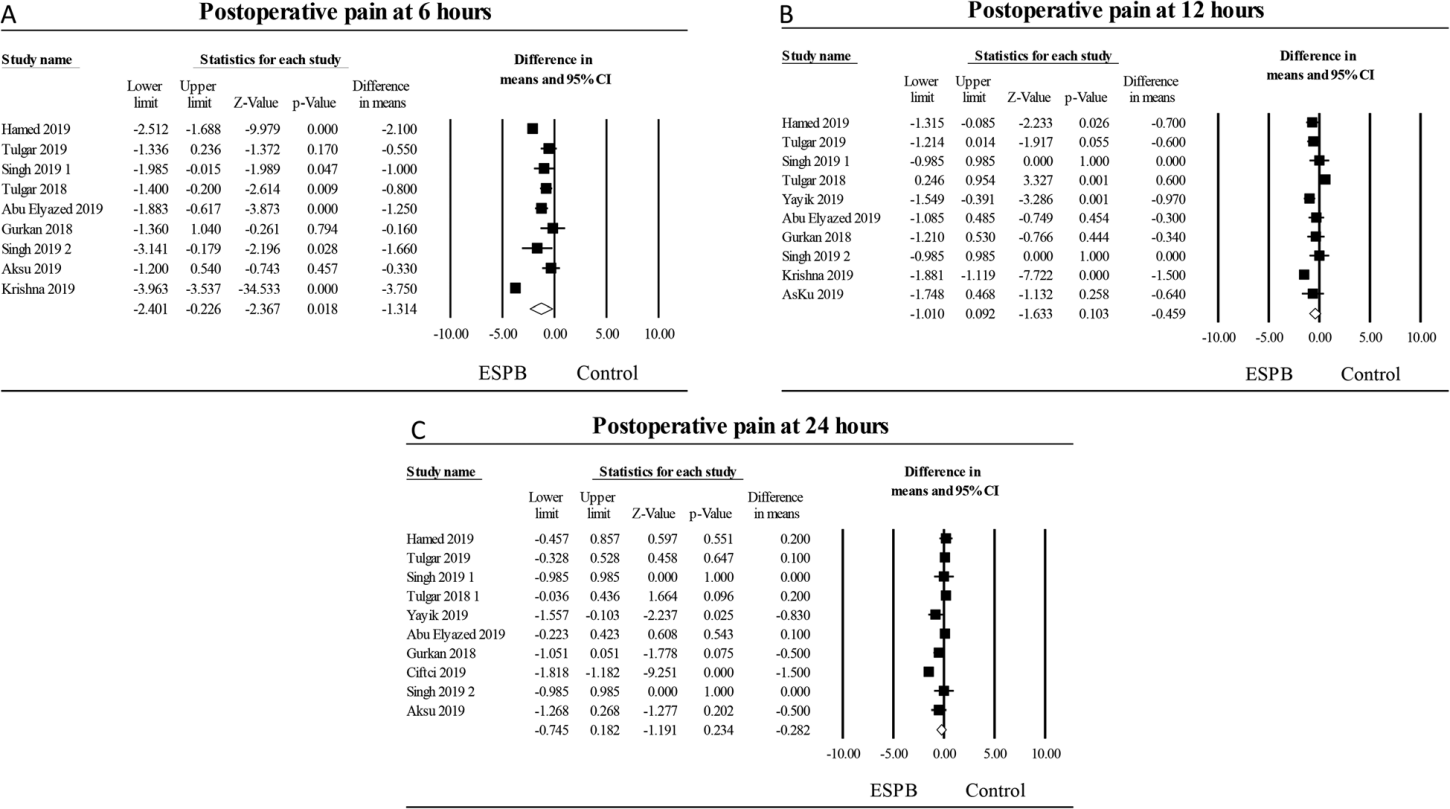
Postoperative pain at rest at 6 h, 12 h and at 24 h. The meta-analysis evaluating the effect of erector spinae plane block on pain scores at 6 h (**a**), at 12 h (**b**), and at 24 h (**c**) compared to control was estimated as a random effect. The point estimate for the overall effect on postoperative pain scores at 6 h following surgery was − 1.31 (95% CI: − 2.40 to − 0.23), *P* < 0.02, (0–10 numerical scale). The point estimate for the overall effect on postoperative pain at 12 h following surgery was − 0.46 (95% CI: − 1.01 to 0.09), *P* = 0.10. The point estimate for the overall effect on postoperative pain scores at 24 h following surgery was − 0.28 (95% CI: − 0.75 to 0.18), *P* = 0.23. The weighted mean difference for individual studies is represented by the square symbol on Forrest plot, with 95% CI of the difference shown as a solid line

The combined effect of nine studies [26, 27, 29–31, 33–35, 37] evaluating erector spinae plane block on postsurgical pain compared to control at 6 h following surgery displayed a significant effect, WMD of − 1.31 (95% CI: − 2.40 to − 0.23) (0–10 numerical scale), *P* < 0.02 (Fig. 3a). Heterogeneity was high (I^2^ = 96%) and could be partially explained by the type of block placement in which the heterogeneity decreased to I^2^ = 89% for studies utilizing single-shot blocks. When investigating the type of surgical procedure the heterogeneity decreased to 10% for studies of spine/orthopedic procedures.

A subgroup analysis looking at type of surgery indicated that the reduction in postsurgical pain compared to control was statistically different in patients who underwent abdominal surgical procedures WMD of − 1.35 (95% CI: − 2.25 to − 0.45), *P* = 0.003, or spine/orthopedic procedures WMD of − 0.95 (95% CI: − 1.60 to − 0.31), *P* = 0.004. However, postsurgical pain compared to control was not different in patients who had chest surgical procedures WMD of − 1.34 (95% CI: − 3.56 to 0.88), *P* = 0.24). A sensitivity analysis was performed by omitting individual studies which did not considerably reduce heterogeneity. An examination of the funnel plot to test publication bias did reveal asymmetry. The Egger’s regression test result was *P* = < 0.001.

### Postoperative pain at activity 6 h after surgery

One study reported the effect of erector spinae plane block on postsurgical pain during movement compared to control at 6 h after surgery and demonstrated a mean difference of − 0.55 (95% CI) -1.21 to 0.11, *P* = 0.01 [37].

### Postoperative pain at rest 12 h following surgery

The effect of ten studies [26, 27, 29–31, 33–35, 37, 38] investigating erector spinae plane block on postoperative surgical pain compared to no block or sham block at 12 h after surgery did not show a significant effect WMD of − 0.46 (95% CI: − 1.01 to 0.09), (0–10 numerical scale), *P* = 0.10, (Fig. 3b). Heterogeneity was found to be high (I^2^ = 87%) and was slightly decreased to I^2^ = 62% for studies using single-shot block placement. The heterogeneity decreased to I^2^ = 0% for studies involving only abdominal surgical procedures. A sensitivity analysis was performed by omitting individual studies which did not significantly reduce heterogeneity.

A subgroup analysis involving the type of surgery revealed that the reduction in postsurgical pain compared to control was statistically different in patients who underwent abdominal surgical procedures WMD of − 0.57 (95% CI: − 0.95 to − 0.19), *P* = 0.003. However, postsurgical pain at rest compared to control was not different in patients 12 h after chest surgical procedures WMD of − 0.70 (95% CI: − 1.51 to 0.12), *P* = 0.09 or spine/orthopedic procedures WMD of − 0.11 (95% CI: − 1.22 to 0.99), *P* = 0.84. An examination of the funnel plot to test publication bias did not reveal asymmetry. The Egger’s test result was *P* = 0.47.

### Postoperative pain at activity 12 h after surgery

There were two studies that reported on postoperative surgical pain at activity 12 h after surgery. Tulgar et al. [37] reported the effect of erector spinae plane block on postsurgical pain during movement compared to control at 12 h after surgery and demonstrated a weighted mean difference of − 0.60 (95% CI: − 1.09 to − 0.11), *P* = 0.02. Yayik et al. [38] reported the effect of erector spinae plane block on postsurgical pain during movement compared to control at 12 h after surgery and demonstrated a weighted mean difference of − 1.14 (95% CI: − 1.50 to − 0.78), *P* < 0.01.

### Postoperative pain at rest 24 h following surgery

The pooled effect of ten studies [26–30, 33–35, 37, 38] examining erector spinae plane block on postoperative surgical pain compared to no block or sham block did not reveal a significant effect WMD of − 0.28 (95% CI: − 0.75 to 0.18), (0–10 numerical scale), *P* = 0.23, (Fig. 3c). Heterogeneity was high (I^2^ = 89%). The heterogeneity decreased to I^2^ = 30% for studies using bilateral block placement. A sensitivity analysis by deleting individual studies did not substantially reduce heterogeneity.

A subgroup analysis involving the type of surgery demonstrated that reduction in postsurgical pain compared to control was not statistically different in patients who underwent abdominal surgical procedures WMD of 0.11 (95% CI: − 0.13 to 0.35), *P* = 0.35, spine/orthopedic surgical procedures WMD of − 0.17 (95% CI: − 0.85 to 0.51), *P* = 0.63, or after chest procedures WMD of − 0.70 (95% CI: − 1.43 to 0.03), *P* < 0.06. An examination of the funnel plot did not reveal asymmetry; Egger’s regression test result was *P* = 0.40.

### Postoperative pain at activity 24 h after surgery

The pooled effect of three studies evaluating the effect of erector spinae plane block on postoperative surgical pain during activity compared to control did not show a significant effect, weighted mean difference of − 0.65 (95% CI: − 1.40 to 0.11), *P* = 0.09. Heterogeneity was I^2^ = 89% [28, 37, 38].

### Postoperative nausea and vomiting (PONV)

**Fig. 4:**
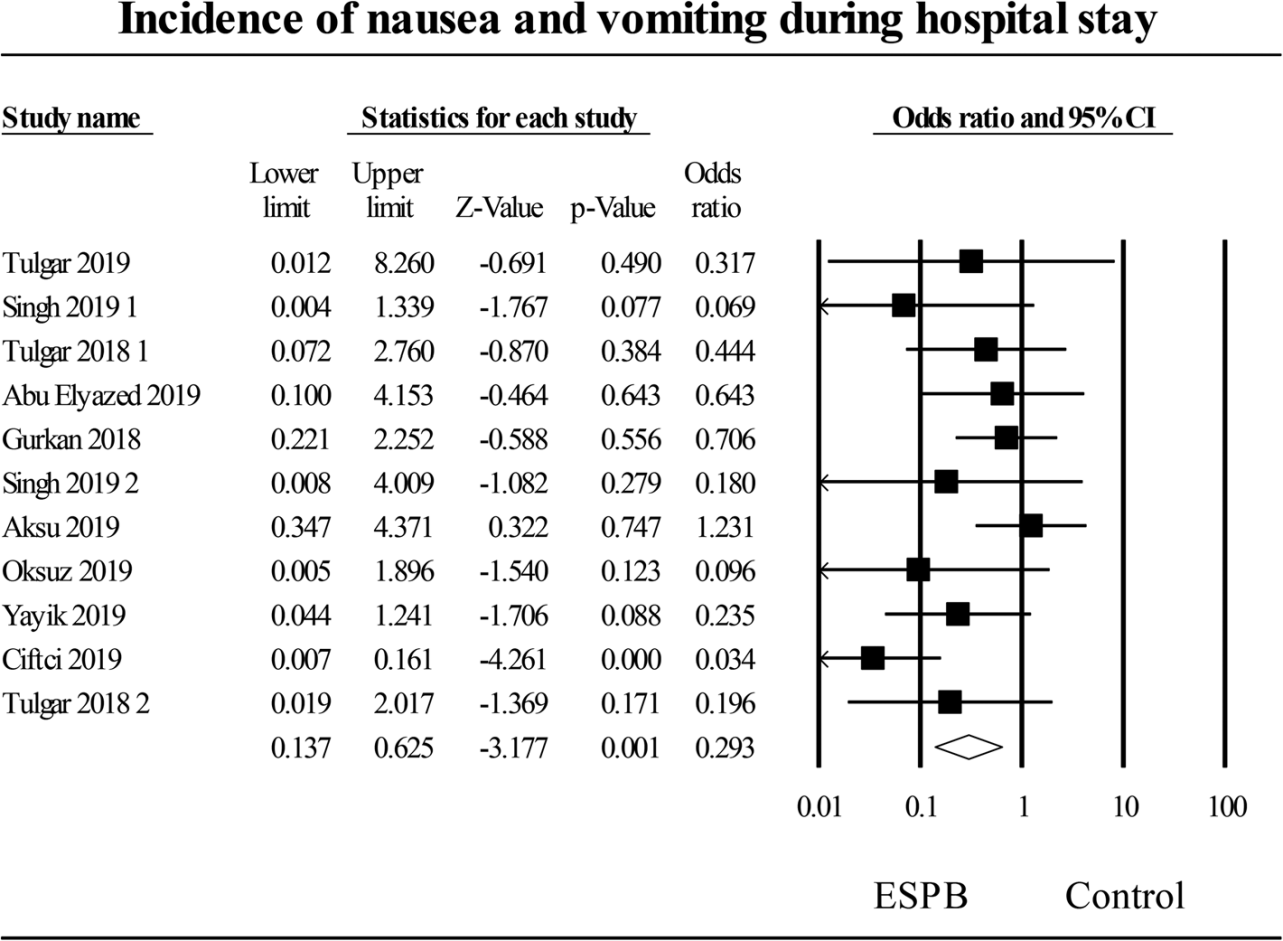
Incidence of postoperative nausea and vomiting at 24 h after surgery. Random-effects meta-analysis evaluating the effect of erector spinae plane block on nausea and vomiting compared to control. Squares to the right of the middle vertical line indicates that erector spinae plane block was associated with increased odds of nausea, whereas squares to the left of the middle vertical line show that erector spinae plane block was associated with decreased odds of nausea. The horizontal lines represent the 95% CI and the diamond shape represents the overall effect of erector spinae plane block on postoperative nausea and vomiting compared to control. CI = confidence interval

The pooled effect of eleven studies [26–29, 31, 33–38] that examined erector spinae plane block on postoperative nausea and vomiting compared to no block or sham block showed a significant effect, OR of 0.29 (95% CI: 0.14 to 0.63) (*P* = 0.001), (Fig. 4). Heterogeneity was moderate, I^2^ = 40%. Heterogeneity was decreased to I^2^ = 0% when investigating either abdominal or spine/orthopedic procedures. A sensitivity analysis by omitting individual studies did not significantly reduce heterogeneity.

A subgroup analysis involving the type of surgery revealed that postoperative nausea and vomiting compared to control was reduced in spine/orthopedic surgical procedures WMD of 0.29 (95% CI: 0.09 to 0.91), *P* = 0.03. In contrast, the postoperative nausea and vomiting compared to no block or sham block was not different in patients who had chest surgery, WMD of 0.22 (95% CI: 0.05 to 1.04), *P* = 0.06 or abdominal surgery WMD of 0.39 (95% CI: 0.10 to 0.1.47), *P* = 0.16.

### Adverse events

All thirteen studies reported either no adverse events (i.e. respiratory depression, local systemic toxicity, hematoma) or did not report any adverse events. One study [26] reported two patients who received erector spinae plane block who experienced intraoperative hypotension compared to one patient in the control group to an estimated incidence of 0.2% (95% CI: 0.3 to 1).

## Discussion

The most important finding of the current investigation was the reduction of postoperative pain in patients who received an erector spinae plane block compared to a control group across multiple surgical procedures. Patients in the erector spinae plane group reported substantially less pain in the immediate postoperative phase (e.g., 6 h after surgery). Our results suggest that the erector spinae plane block is an effective strategy to reduce postsurgical pain.

Our results are important as pain continues to be poorly controlled after surgery. A recent study by Herbst et al. showed that 23.3% of postsurgical readmissions were related to poor postoperative pain control [39]. Appropriate postoperative analgesia control has been associated with improved patient satisfaction, and it is utilized in the HCAPS survey used to evaluate quality of care in hospitals [40, 41]. Thus, by using the erector spinae plane block, clinical practitioners may reduce pain-related readmissions and improve patient satisfaction after surgery.

Another important finding of our current investigation was the effect of the erector spinae plane block on the reduction of postoperative nausea and vomiting. This is interesting as not all analgesic interventions have been shown to reduce opioid-related side effects [42–44]. In addition, the effect was large and comparable to other first line pharmacological agents for postoperative nausea and vomiting prophylaxis. Based on our results, one could argue that the effect of the erector spinae plane block on PONV was likely due to the reduction of postoperative pain rather than the estimated opioid sparing effects.

One of the main advantages of the erector spinae plane block is that the block is considered easy to be performed, especially when compared to paravertebral blocks or thoracic epidurals. This is important because it not only maximizes efficacy of the block, but also allows its implementation across multiple surgical procedures. The injection is performed deep in the erector spinae muscle and superficial to the tips of the thoracic transverse processes. The block has an excellent safety profile since the local anesthetic injection is distant from the pleura, major blood vessels, and spinal cord.

The anatomical localization of the spinal nerves and the different anatomy of the vertebral column may be a major factor for the various postoperative outcomes following the placement of an erector spinae plane block. Recent literature has reported that different volumes of local anesthetic injectate and its corresponding spread are influenced by the site of injection. For example, a 5 mL of injectate was needed to cover one vertebral level in the lumbar region, whereas only 3.3 (radiological imaging studies) to 3.5 (cadaveric dissections studies) mL are needed in thoracic region [45]. In our study, we found that patients who underwent spine or orthopedic surgeries compared to control experienced clinical pain relief at 6 h which dissipated by 12 h after surgery. In contrast, studies investigating erector spinae plane block to control in patients undergoing chest surgical procedures reported no significant pain relief at any three of the study time periods in the postoperative period. Nonetheless, patients who underwent chest or spine/orthopedic procedures reported opioid sparing effects at 24 h after surgery. Future clinical trials investigating the optimal volume of local anesthetics in different anatomical regions and different types of surgeries to determine analgesic adequacy is warranted.

The findings of our study should only be interpreted within the context of its limitations. First, in order to minimize heterogeneity, we compared erector spinae plane block to an “inactive” control group. More recently, studies have compared the erector spinae plane blocks to other commonly performed blocks (e.g., transversus abdominis plane block, paravertebral blocks) [46–48]. Nonetheless, the number of randomized trials are not yet adequate to perform a quantitative analysis comparing the erector spinae plane blocks to other regional blocks. Secondly, we limited our comparison to acute postoperative pain. Some recent reports have highlighted the potential use of the erector spinae plane block for chronic pain conditions [49, 50]. It is conceivable that the erector spinae plane block may reduce opioid consumption among chronic pain patients. Third, we did not include studies investigating continuous catheter infusions of local anesthetics in the erector spine plane as most investigations are limited to case reports. The use of a continuous catheter erector spinae block can prolong the local anesthetic blockade extending the postoperative pain relief beyond 12 h [51, 52]. Randomized trials confirming the efficacy of continuous catheter erector spinae blocks are warranted due to the limited analgesic duration of single-shot blocks. Last, we included a large multitude of surgical procedures with various anatomical differences in an attempt to improve the generalizability of our findings, which may account for the significant heterogeneity present in the current studies. Nonetheless, we used the random effect model for all of the analyses and were able to explain some of the heterogeneity based on the utilization of either unilateral or bilateral placement of the block or by the category of surgical location. However, the high levels of heterogeneity among the studies makes publication bias concerning in the studies published to date. Further investigations of erector spinae plane block for postoperative analgesia with larger sample sizes are needed to address the wide variability of the effect sizes seen in our analysis.

## Conclusion

In summary, our results provide moderate-quality evidence the erector spinae plane block may be an effective analgesic strategy to minimize postoperative pain and reduce postoperative opioid consumption across several types of surgeries. In addition, a high quality of evidence demonstrated that erector spinae plane block also reduced postoperative nausea and vomiting. More studies are necessary to confirm our findings of a possible short-term analgesic benefit of the erector spinae plane block.

## Supplementary information

**Additional file 1.** Search strategy.

## Data Availability

The datasets generated and analyzed during the current study are available from the corresponding author on reasonable request.

## Abbreviations

CI: Confidence interval
ESPB: Erector spinae plane block
GRADE: Grading of Recommendations, Assessment, Development, and Evaluation
HCAPS: Hospital Consumer Assessment of Healthcare Providers and Systems
I^2^: Heterogeneity
IQR: Interquartile range
IV: Intravenous
NRS: Numrical rating scale
WMD: Weighted mean differences

## References

[1] Neuman MD, Bateman BT, Wunsch H (2019). Inappropriate opioid prescription after surgery. Lancet.

[2] Beloeil H, Albaladejo P, Sion A (2019). Multicentre, prospective, double-blind, randomised controlled clinical trial comparing different non-opioid analgesic combinations with morphine for postoperative analgesia: the OCTOPUS study. Br J Anaesth.

[3] Soffin EM, Lee BH, Kumar KK, Wu CL (2019). The prescription opioid crisis: role of the anesthesiologist in reducing opioid use and misuse. Br J Anaesth.

[4] Yao Y, Li J, Hu H, Xu T, Chen Y (2019). Ultrasound-guided serratus plane block enhances pain relief and quality of recovery after breast cancer surgery: A randomised controlled trial. Eur J Anaesthesiol.

[5] Rao Kadam V, Ludbrook G, van Wijk RM (2019). Comparison of ultrasound-guided transmuscular quadratus lumborum block catheter technique with surgical pre-peritoneal catheter for postoperative analgesia in abdominal surgery: a randomised controlled trial. Anaesthesia.

[6] Clement JC, Besch G, Puyraveau M, et al. Clinical Effectiveness of single dose of intravenous dexamethasone on the duration of ropivacaine axillary brachial plexus block: the randomized placebo-controlled ADEXA trial. Reg Anesth Pain Med. 2019;44:370-4. 10.1136/rapm-2018-100035. 10.1136/rapm-2018-100035 30777900

[7] Lovett-Carter D, Kendall MC, McCormick ZL, et al. Pectoral nerve blocks and postoperative pain outcomes after mastectomy: a meta-analysis of randomized controlled trials. Reg Anesth Pain Med. 2019. 10.1136/rapm-2019-100658. 10.1136/rapm-2019-100658 31401620

[8] Schnabel A, Reichl SU, Weibel S (2018). Efficacy and safety of dexmedetomidine in peripheral nerve blocks: A meta-analysis and trial sequential analysis. Eur J Anaesthesiol.

[9] Mayhew D, Sahgal N, Khirwadkar R, Hunter JM, Banerjee A (2018). Analgesic efficacy of bilateral superficial cervical plexus block for thyroid surgery: meta-analysis and systematic review. Br J Anaesth.

[10] Taketa Y, Irisawa Y, Fujitani T. Comparison of ultrasound-guided erector spinae plane block and thoracic paravertebral block for postoperative analgesia after video-assisted thoracic surgery: a randomized controlled non-inferiority clinical trial. Reg Anesth Pain Med. 2019. 10.1136/rapm-2019-100827. 10.1136/rapm-2019-100827 31704789

[11] Adhikary SD, Liu WM, Fuller E, Cruz-Eng H, Chin KJ (2019). The effect of erector spinae plane block on respiratory and analgesic outcomes in multiple rib fractures: a retrospective cohort study. Anaesthesia.

[12] Moore RP, Liu CJ, George P, et al. Early experiences with the use of continuous erector spinae plane blockade for the provision of perioperative analgesia for pediatric liver transplant recipients. Reg Anesth Pain Med. 2019. 10.1136/rapm-2018-100253. 10.1136/rapm-2018-100253 30992412

[13] Hamadnalla H, Elsharkawy H, Shimada T, Maheshwari K, Esa WAS, Tsui BCH (2019). Cervical erector spinae plane block catheter for shoulder disarticulation surgery. Can J Anaesth.

[14] Tulgar S, Selvi O, Kapakl MS (2018). Erector spinae plane block for different laparoscopic abdominal surgeries: case series. Case Rep Anesthesiol.

[15] Forero M, Adhikary SD, Lopez H, Tsui C, Chin KJ (2016). The Erector Spinae Plane Block: A Novel Analgesic Technique in Thoracic Neuropathic Pain. Reg Anesth Pain Med.

[16] Chin KJ, Malhas L, Perlas A (2017). The Erector Spinae Plane Block Provides Visceral Abdominal Analgesia in Bariatric Surgery: A Report of 3 Cases. Reg Anesth Pain Med.

[17] Moher D, Liberati A, Tetzlaff J, Altman DG, PRISMA Group (2009). Preferred reporting items for systematic reviews and meta-analyses: the PRISMA statement. PLoS Med.

[18] Kendall MC, Castro Alves LJ, De Oliveira G (2018). Liposome bupivacaine compared to plain local anesthetics to reduce postsurgical pain: an updated meta-analysis of randomized controlled trials. Pain Res Treat.

[19] Kendall MC, Alves LJ, Pence K, Mukhdomi T, Croxford D, De Oliveira GS (2020). The effect of intraoperative methadone compared to morphine on postsurgical pain: a meta-analysis of randomized controlled trials. Anesthesiol Res Pract.

[20] Available: http://www.globalrph.com/narcoticonv.htm [Accessed Last accessed 7/2019]..

[21] Wan X, Wenqian W, Liu J, Tong T (2014). Estimating the sample mean and standard deviation from the sample size, median, Range And/or Interquartile Range. BMC Med Res Methodol.

[22] Hozo SP, Djulbegovic B, Hozo I (2005). Estimating the mean and variance from the median, range, and the size of a sample. BMC Med Res Methodol.

[23] Sterne JAC, Savović J, Page MJ (2019). RoB 2: a revised tool for assessing risk of bias in randomised trials. BMJ.

[24] DerSimonian R, Laird N (1986). Meta-analysis in clinical trials. Control Clin Trials.

[25] Egger M, Davey Smith G, Schneider M (1997). Bias in meta-analysis detected by a simple, graphical test. BMJ.

[26] Abu Elyazed MM, Mostafa SF, Abdelghany MS, Eid GM (2019). Ultrasound-guided erector spinae plane block in patients undergoing open epigastric hernia repair: a prospective randomized controlled study. Anesth Analg.

[27] Aksu C, Kus A, Yorukoglu HU, Kilic CT, Gurkan Y (2019). Analgesic effect of the bi-level injection erector spinae plane block after breast surgery: a randomized controlled trial. Agri.

[28] Ciftci B, Aksoy M, Ince I, Ahıskalıoglu A, Yılmazel UE (2018). The effects of positive end-expiratory pressure at different levels on postoperative respiration parameters in patients undergoing laparoscopic cholecystectomy. J Investig Surg.

[29] Gürkan Y, Aksu C, Kuş A, Yörükoğlu UH, Kılıç CT (2018). Ultrasound guided erector spinae plane block reduces postoperative opioid consumption following breast surgery: a randomized controlled study. J Clin Anesth.

[30] Hamed MA, Goda AS, Basiony MM, Fargaly OS, Abdelhady MA (2019). Erector spinae plane block for postoperative analgesia in patients undergoing total abdominal hysterectomy: a randomized controlled study original study. J Pain Res.

[31] Krishna SN, Chauhan S, Bhoi D (2019). Bilateral erector spinae plane block for acute post-surgical pain in adult cardiac surgical patients: a randomized controlled trial. J Cardiothorac Vasc Anesth.

[32] Oksuz G, Bilgen F, Arslan M, Duman Y, Urfalıoglu A, Bilal B (2019). Ultrasound-guided bilateral erector spinae block versus tumescent anesthesia for postoperative analgesia in patients undergoing reduction mammoplasty: a randomized controlled study. Aesthet Plast Surg.

[33] Singh S, Kumar G, Akhileshwar (2019). Ultrasound-guided erector spinae plane block for postoperative analgesia in modified radical mastectomy: A randomised control study. Indian J Anaesth.

[34] Singh S, Choudhary NK, Lalin D, Verma VK. Bilateral ultrasound-guided erector spinae plane block for postoperative analgesia in lumbar spine surgery: a randomized control trial. J Neurosurg Anesthesiol. 2019. 10.1097/ANA.0000000000000603. 10.1097/ANA.0000000000000603 31033625

[35] Tulgar S, Kapakli MS, Senturk O, Selvi O, Serifsoy TE, Ozer Z (2018). Evaluation of ultrasound-guided erector spinae plane block for postoperative analgesia in laparoscopic cholecystectomy: a prospective, randomized, controlled clinical trial. J Clin Anesth.

[36] Tulgar S, Kose HC, Selvi O (2018). Comparison of ultrasound-guided lumbar erector spinae plane block and Transmuscular Quadratus Lumborum block for postoperative analgesia in hip and proximal femur surgery: a prospective randomized feasibility study. Anesth Essays Res.

[37] Tulgar S, Kapakli MS, Kose HC (2019). Evaluation of ultrasound-guided erector spinae plane block and oblique subcostal transversus abdominis plane block in laparoscopic cholecystectomy: randomized, controlled, Prospective Study. Anesth Essays Res.

[38] Yayik AM, Cesur S, Ozturk F (2019). Postoperative analgesic efficacy of the ultrasound-guided erector spinae plane block in patients undergoing lumbar spinal decompression surgery: a randomized controlled study. World Neurosurg.

[39] Herbst MO, Price MD, Soto RG (2017). Pain related readmissions /revisits following same-day surgery: Have they decreased over a decade?. J Clin Anesth.

[40] Shanthanna H, Paul J, Lovrics P (2019). Satisfactory analgesia with minimal emesis in day surgeries: a randomised controlled trial of morphine versus hydromorphone. Br J Anaesth.

[41] Smith GA, Chirieleison S, Levin J (2019). Impact of length of stay on HCAPS scores following lumbar spine surgery. J Neurosurg Spine.

[42] Fujii T, Shibata Y, Akane A (2019). A randomised controlled trial of pectoral nerve-2 (PECS 2) block vs. serratus plane block for chronic pain after mastectomy. Anaesthesia.

[43] De Oliveira GS, Castro Alves LJ, Nader A, Kendall MC, Rahangdale R, McCarthy RJ (2014). Perineural dexamethasone to improve postoperative analgesia with peripheral nerve blocks: a meta-analysis of randomized controlled trials. Pain Res Treat.

[44] Gasanova I, Alexander JC, Estrera K (2019). Ultrasound-guided suprainguinal fascia iliaca compartment block versus periarticular infiltration for pain management after total hip arthroplasty: a randomized controlled trial. Reg Anesth Pain Med.

[45] De Cassai A, Andreatta G, Bonvicini D, Boscolo A, Munari M, Navalesi P (2020). Injectate spread in ESP block: a review of anatomical investigations. J Clin Anesth.

[46] Nagaraja PS, Ragavendran S, Singh NG (2018). Comparison of continuous thoracic epidural analgesia with bilateral erector spinae plane block for perioperative pain management in cardiac surgery. Ann Card Anaesth.

[47] Heinink T (2019). Erector spinae block or paravertebral block or thoracic epidural for analgesia after rib fracture?. Anaesthesia..

[48] Wang HJ, Liu Y, Ge WW (2019). Comparison of ultrasound-guided serratus anterior plane block and erector spinae plane block perioperatively in radical mastectomy. Zhonghua Yi Xue Za Zhi.

[49] Tulgar S, Selvi O, Senturk O, Serifsoy TE, Thomas DT (2019). Ultrasound-guided erector spinae plane block : Indications, Complications, and Effects on Acute and Chronic Pain Based on a Single-center Experience. Cureus.

[50] Kot Baixauli P, Rodriguez Gimillo P, Baldo Gosalvez J, De Andrés Ibáñez J (2019). The erector spinae plane block (ESPB) in the management of chronic thoracic pain. Correlation of pain/analgesia areas and long-term effect of the treatment in three cases. Rev Esp Anestesiol Reanim.

[51] Forero M, Rajarathinam M, Adhikary S, Chin KJ (2017). Continuous erector spinae plane block for rescue analgesia in thoracotomy after epidural failure: a case report. A Case Rep.

[52] Scimia P, Basso Ricci E, Droghetti A, Fusco P (2017). The ultrasound-guided continuous erector spinae plane block for postoperative analgesia in video-assisted thoracoscopic lobectomy. Reg Anesth Pain Med.

