# Supplementary material for: The effect of ultrasound-guided erector spinae plane block on postsurgical pain: a meta-analysis of randomized controlled trials
Source: BMC Anesthesiol. 2020 May 1;20:99. doi: 10.1186/s12871-020-01016-8 (PMC7195766; doi:10.1186/s12871-020-01016-8)
Supplement: Supplementary file 1 — Additional file 1. Search strategy. [file 12871_2020_1016_MOESM1_ESM.docx]

**Additional file 1**

Search strategy

PubMed Search = 544

(erector spinae plane block[Title] OR (erector[All Fields] AND spinae[All Fields] AND block[All Fields])) OR ESPB[All Fields]

Cochrane Database of Systematic Reviews = 208

erector spinae plane block in Title Abstract Keyword OR erector spine block in Title Abstract Keyword OR ESPB in Title Abstract Keyword

Embase = 35

'erector spinae plane block'/exp OR 'erector spinae plane block' OR 'erector spinae block':jt OR espb:au AND randomized controlled trial

Google Scholar = 116

'erector spinae plane block' or erector spinae block
